# Unmasking Granulomatosis With Polyangiitis Through a Large Pulmonary Cavity: A Case Report and Review of the Literature

**DOI:** 10.7759/cureus.113176

**Published:** 2026-07-22

**Authors:** Abdirahman Ahmed, Regene T Mosely, Lakisha B Willis, Yousef Alsmairat, Hawazin Abbas

**Affiliations:** 1 Internal Medicine, Michigan State University, Lansing, USA; 2 Internal Medicine, Authority Health, Detroit, USA; 3 Radiology, Trinity Health Oakland Hospital, Pontiac, USA; 4 Internal Medicine, Wayne State University School of Medicine, Detroit, USA; 5 Pulmonology, Trinity Health Oakland Hospital, Pontiac, USA

**Keywords:** antineutrophil cytoplasmic antibody (anca) associated vasculitis (aav), cavitary lung lesions, granulomatosis with polyangiitis (gpa), proteinase 3 (pr3)-positive granulomatosis with polyangiitis (gpa), true hemoptysis

## Abstract

Granulomatosis with polyangiitis (GPA) is an antineutrophil cytoplasmic antibody (ANCA)-associated vasculitis characterized by necrotizing inflammation of small- and medium-sized vessels. Although pulmonary involvement is common, isolated pulmonary presentations without upper airway or renal manifestations are uncommon and may mimic infection or malignancy, resulting in diagnostic delays.

A 33-year-old male presented with four days of hemoptysis accompanied by fever, chills, unintentional weight loss, and bilateral ankle and knee arthralgias. Chest imaging demonstrated a large 7 × 8 × 8 cm thick-walled cavitary lesion in the right lower lobe. Given the radiographic appearance, an extensive infectious workup was pursued and proved negative, including evaluation for bacterial, fungal, viral, and mycobacterial pathogens. Autoimmune serologies revealed positive cytoplasmic antineutrophil cytoplasmic antibody (C-ANCA) and elevated proteinase-3 (PR3) antibodies. Bronchoscopy with transbronchial biopsy demonstrated nonspecific inflammatory changes without evidence of necrotizing granulomatous inflammation. Despite nondiagnostic histopathology, the combination of clinical presentation, radiographic findings, and highly specific serologic markers supported the diagnosis of GPA. The patient was treated with high-dose corticosteroids and rituximab, resulting in significant clinical improvement.

This case highlights the diagnostic challenges of isolated pulmonary GPA presenting as cavitary lung disease. It emphasizes the importance of maintaining a broad differential diagnosis when evaluating cavitary pulmonary lesions and demonstrates the diagnostic value of ANCA serologies when tissue sampling is inconclusive. Early recognition and initiation of immunosuppressive therapy are critical to preventing disease progression and irreversible organ damage.

## Introduction

Granulomatosis with polyangiitis (GPA), formerly known as Wegener granulomatosis, is a rare antineutrophil cytoplasmic antibody (ANCA)-associated vasculitis characterized by necrotizing granulomatous inflammation of small- and medium-sized blood vessels [1]. The disease most commonly affects the upper respiratory tract, lungs, and kidneys, with pulmonary involvement occurring in up to 85% of patients [2]. Pulmonary manifestations include nodules, infiltrates, diffuse alveolar hemorrhage, and cavitary lesions [2]. Although cavitary pulmonary lesions are reported in approximately one-quarter of patients with GPA, isolated pulmonary presentations without concomitant otolaryngologic or renal involvement are uncommon and may create significant diagnostic uncertainty [2].

The differential diagnosis of cavitary lung disease is broad and includes bacterial, mycobacterial, and fungal infections, septic emboli, primary pulmonary malignancies, and other inflammatory disorders [2]. Consequently, patients often undergo extensive evaluations before an autoimmune etiology is considered. While histopathologic confirmation remains the diagnostic gold standard, tissue sampling may be limited by lesion location or nondiagnostic findings, making serologic testing an important component of diagnosis [1]. Cytoplasmic ANCA (C-ANCA) directed against proteinase 3 (PR3) demonstrates high specificity for GPA and may be particularly useful when clinical suspicion remains high despite inconclusive biopsy results [1,2].

We report a case of GPA presenting as a large cavitary pulmonary lesion in a young male without classic ear, nose, throat, or renal manifestations. This case highlights the importance of maintaining a broad differential diagnosis for cavitary lung lesions and illustrates the diagnostic value of integrating clinical, radiographic, and serologic findings when histopathology is nondiagnostic.

## Case presentation

A 33-year-old Caucasian male presented with four days of hemoptysis associated with approximately one month of fever, chills, unintentional weight loss, and bilateral ankle and knee pain that worsened with movement. He denied dyspnea, chest pain, nasal congestion, sinus symptoms, epistaxis, dysuria, hematuria, or other urinary complaints. He had no known history of autoimmune disease and denied recent travel, tuberculosis exposure, or other significant infectious risk factors.

On presentation, physical examination was notable for mild bilateral ankle and knee tenderness without obvious joint deformity or swelling. Pulmonary examination revealed no significant wheezing or crackles. The remainder of the examination was unremarkable, including the absence of upper airway findings suggestive of sinonasal involvement.

Initial laboratory evaluation demonstrated marked systemic inflammation, including elevated erythrocyte sedimentation rate (101 mm/hr) and C-reactive protein (14.9 mg/dL). Autoimmune testing revealed positive proteinase-3 (PR3) antibodies and a positive C-ANCA titer, while extensive infectious evaluation was unrevealing (Table 1).

**Table 1:** Initial laboratory evaluation

Laboratory test	Result	Units	Reference range
White blood cell count	11.0	×10³/µL	4.0–11.0
Hemoglobin	11.8	g/dL	13.5–17.5
Platelet count	536	×10³/µL	150–400
Blood urea nitrogen	13	mg/dL	7–20
Creatinine	0.73	mg/dL	0.6–1.3
Erythrocyte sedimentation rate (ESR)	101	mm/hr	0–10
C-reactive protein (CRP)	14.9	mg/dL	<0.5
Antinuclear antibody (ANA)	Negative	—	Negative
Rheumatoid factor	45	IU/mL	<14
Myeloperoxidase antibody (MPO)	<0.2	U/mL	Negative
Proteinase-3 antibody (PR3)	33.0	U/mL	Negative
C-ANCA titer	1:80	—	Negative
P-ANCA titer	<1:20	—	Negative
HIV Ag/Ab	Nonreactive	—	Nonreactive
QuantiFERON-TB Gold Plus	Negative	—	Negative
Histoplasma urine antigen	<0.2	ng/mL	Negative
Blastomyces antibody	Not detected	—	Negative
Coccidioides antibody	Not detected	—	Negative
Histoplasma antibody	Not detected	—	Negative
Aspergillus antibody	Not detected	—	Negative
Aspergillus antigen index	<0.50	Index	Negative

**Figure 1:**
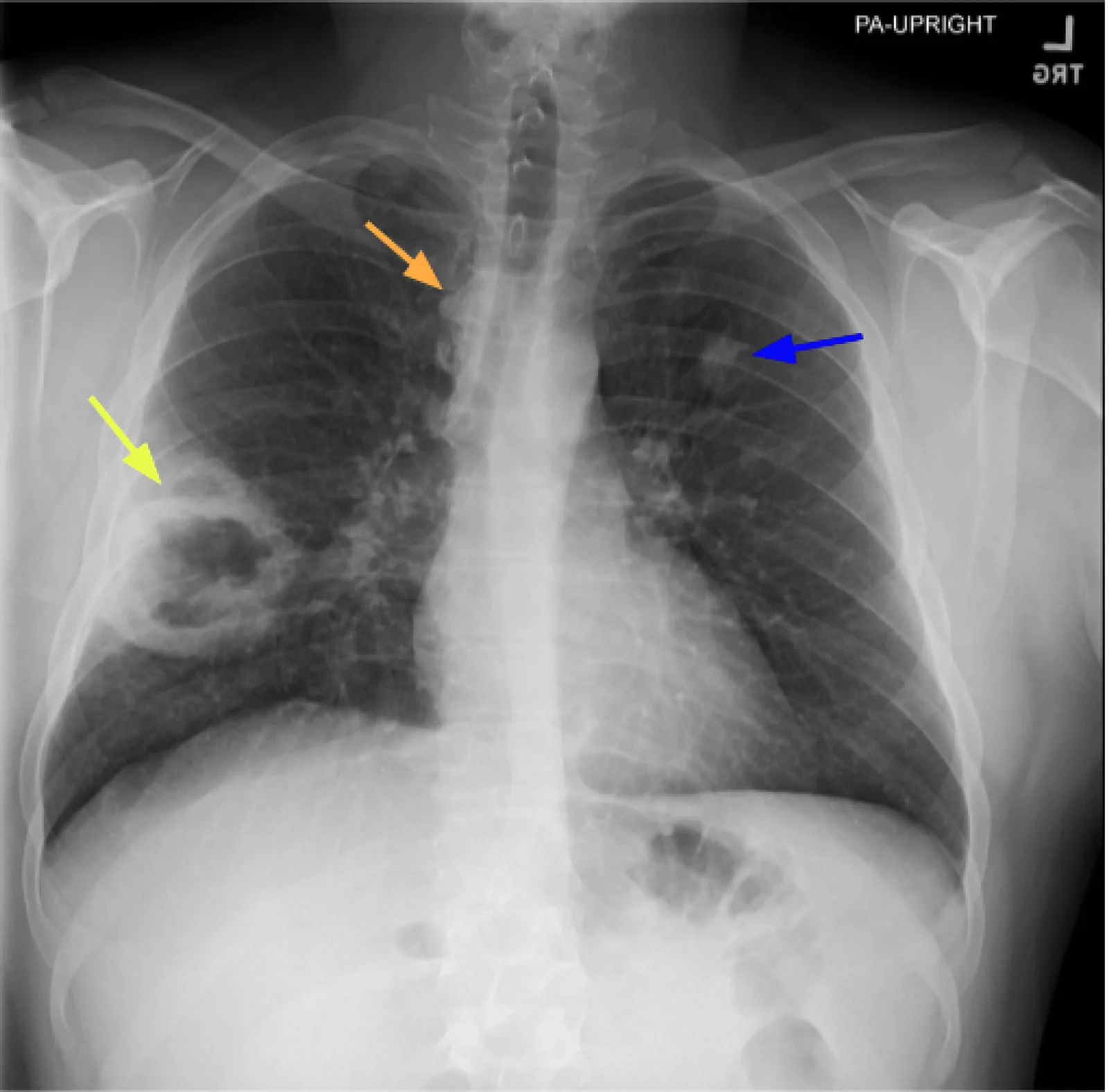
Posteroanterior chest radiograph demonstrating a large cavitary lesion centered within the superior segment of the right lower lobe, with possible extension toward the right middle lobe (yellow arrow). Additional small focal nodular opacities are present in the right upper lobe (orange arrow) and left upper lobe (blue arrow).

Chest radiography revealed a large cavitary lesion within the superior segment of the right lower lobe and a smaller focal opacity in the left upper lobe (Figure 1).

**Figure 2:**
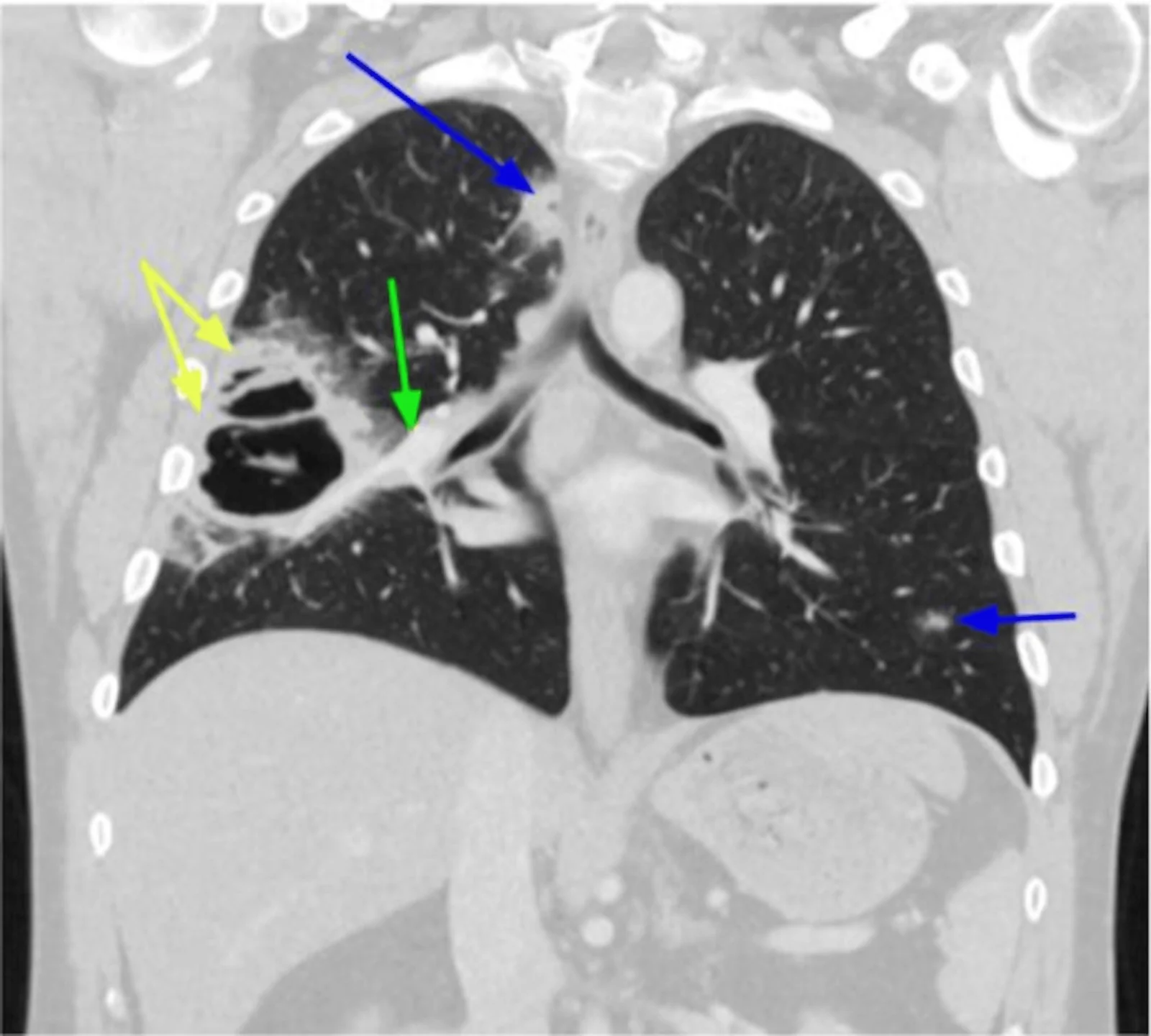
Coronal CT chest showing a large thick-walled irregular cavitary lesion in the superior segment of the right lower lobe extending into the lateral segment of the right middle lobe and inferior aspect of the right upper lobe with adjacent opacities (yellow arrows). Extension of this process into the right hilum (green arrow) and scattered noncalcified solid pulmonary nodules (blue arrows), consistent with granulomatosis with polyangiitis.

Subsequent computed tomography (CT) of the chest demonstrated a thick-walled cavitary lesion measuring approximately 7 × 8 × 8 cm with surrounding nodularity and multifocal pulmonary opacities (Figure 2). Given the radiographic appearance, infectious etiologies were initially considered most likely.

An extensive infectious evaluation was performed. Testing for human immunodeficiency virus, hepatitis B virus, hepatitis C virus, methicillin-resistant *Staphylococcus aureus*, *Bordetella pertussis*, *Chlamydia pneumoniae*, tuberculosis, and invasive fungal infection was negative. Acid-fast bacilli cultures, QuantiFERON-TB testing, and serum beta-D-glucan were unrevealing.

Given the negative infectious workup and associated constitutional symptoms, autoimmune serologies were obtained. Cytoplasmic antineutrophil cytoplasmic antibody (C-ANCA) was positive at a titer of 1:80, and proteinase-3 (PR3) antibody levels were elevated at 33.0 U/mL. Antinuclear antibody (ANA), perinuclear ANCA (P-ANCA), anti-myeloperoxidase (MPO), and anti-double-stranded DNA antibodies were negative.

Pulmonology and infectious disease specialists were consulted. Bronchoscopy with bronchoalveolar lavage and transbronchial biopsy was performed. Microbiologic studies and cytology were negative. Histopathologic examination demonstrated nonspecific inflammatory changes without evidence of malignancy, vasculitis, or necrotizing granulomatous inflammation.

Despite nondiagnostic tissue sampling, the combination of constitutional symptoms, cavitary pulmonary disease, positive C-ANCA, and elevated PR3 antibodies strongly supported a diagnosis of granulomatosis with polyangiitis. The patient was initiated on high-dose corticosteroid therapy and rituximab for remission induction. Trimethoprim-sulfamethoxazole was prescribed for Pneumocystis jirovecii prophylaxis.

Following treatment initiation, the patient experienced significant improvement in hemoptysis and constitutional symptoms. He was discharged in stable condition with close rheumatology and pulmonology follow-up for ongoing monitoring and maintenance therapy planning.

## Discussion

Granulomatosis with polyangiitis (GPA) is a rare antineutrophil cytoplasmic antibody (ANCA)-associated vasculitis characterized by necrotizing granulomatous inflammation and small- to medium-vessel vasculitis. Although the respiratory tract and kidneys are the most commonly affected organs, the clinical presentation is highly variable and may result in significant diagnostic delays. Pulmonary involvement occurs in up to 85% of patients and may manifest as nodules, infiltrates, diffuse alveolar hemorrhage, tracheobronchial disease, or cavitary lesions [2]. While cavitary pulmonary lesions have been reported in approximately one-quarter of patients with GPA, isolated pulmonary presentations without concurrent upper airway or renal involvement are considerably less common [2].

The differential diagnosis of cavitary lung lesions is broad and frequently favors infectious or malignant etiologies. Common considerations include tuberculosis, fungal infections, necrotizing bacterial pneumonia, septic emboli, primary lung malignancy, and metastatic disease. In our patient, the presence of hemoptysis, constitutional symptoms, and a large cavitary lesion initially raised concern for an infectious process. However, an extensive evaluation for bacterial, mycobacterial, fungal, and viral pathogens was unrevealing, prompting further investigation into autoimmune causes. This case illustrates the importance of maintaining a broad differential diagnosis when evaluating cavitary pulmonary lesions, particularly in younger patients without clear infectious risk factors.

Serologic testing played a pivotal role in establishing the diagnosis. Cytoplasmic ANCA directed against proteinase 3 (PR3) is highly specific for GPA and remains a cornerstone of diagnosis when histopathologic findings are inconclusive. Studies have reported specificities exceeding 95% in patients with active disease [1,2]. Furthermore, PR3-ANCA positivity has been associated with an increased risk of relapse compared with MPO-ANCA-positive disease [3]. The strong serologic profile in our patient, combined with characteristic clinical and radiographic findings, provided compelling evidence supporting the diagnosis despite nondiagnostic tissue sampling.

Histopathologic confirmation remains the diagnostic gold standard for GPA; however, biopsy sensitivity varies substantially depending on the site and method of sampling. Bronchoscopic and transbronchial sampling may be nondiagnostic because of limited tissue acquisition and sampling error. In our patient, mediastinal lymph node aspiration was negative for malignant cells, while lung biopsy specimens yielded only benign respiratory epithelium without diagnostic evidence of vasculitis, granulomatous inflammation, or malignancy. Consequently, a nondiagnostic biopsy should not exclude GPA when the overall clinical, serologic, and radiographic picture remains strongly suggestive. Consistent with the 2022 American College of Rheumatology/European Alliance of Associations (ACR/EULAR) classification criteria, the diagnosis of GPA relies on integration of clinical, serologic, radiographic, and histopathologic findings rather than any single diagnostic modality [1].

The treatment landscape for GPA has evolved considerably over the past two decades. High-dose glucocorticoids remain a cornerstone of remission induction, while rituximab has emerged as a preferred therapeutic agent for many patients. The Rituximab in ANCA-Associated Vasculitis (RAVE) trial demonstrated that rituximab was non-inferior to cyclophosphamide for induction of remission [4] and may be particularly beneficial in patients with relapsing disease. Similarly, the Rituximab versus Cyclophosphamide in ANCA-Associated Renal Vasculitis (RITUXVAS) trial confirmed the efficacy of rituximab in severe ANCA-associated vasculitis [5]. For maintenance therapy, the Maintenance of Remission using Rituximab in Systemic ANCA-Associated Vasculitis (MAINRITSAN) trial demonstrated lower relapse rates with rituximab compared with azathioprine [6]. These findings have informed contemporary treatment guidelines, and current EULAR recommendations support the use of rituximab for remission induction and maintenance in appropriately selected patients [7]. Given our patient's PR3 positivity and the known association between PR3 antibodies and relapse risk, rituximab represented an appropriate therapeutic strategy.

Review of previously published reports demonstrates that cavitary pulmonary GPA frequently mimics infectious or malignant processes, resulting in delayed diagnosis. Several reported cases were initially treated as pulmonary abscesses, tuberculosis, or necrotizing pneumonia before vasculitis was recognized [8-11] (Table 2). Similar to our patient, cases of limited or isolated pulmonary GPA without renal involvement have been described, although such presentations remain uncommon. A recurring theme across published reports is the reliance on serologic testing and radiographic findings when tissue sampling is nondiagnostic. Our case further contributes to the literature by illustrating a large cavitary lesion as the predominant manifestation of GPA in the absence of classic upper airway or renal disease, highlighting the importance of maintaining vasculitis in the differential diagnosis of cavitary lung lesions.

**Table 2:** Comparison of selected published cases of GPA presenting with cavitary pulmonary disease. GPA, granulomatosis with polyangiitis; NR, not reported; M, male; F, female.

Author (reference)	Year	Age/sex	Presentation	ANCA status	Histopathology	Treatment	Outcome
Lakhani et al. [8]	2021	NR/M	Cavitary pulmonary lesion	C-ANCA+/PR3+	Diagnostic	Steroids + rituximab	Improved
Martins et al. [9]	2024	NR/F	Initially treated as tuberculosis	C-ANCA+	Diagnostic	Immunosuppression	Improved
Iyer et al. [10]	2021	Adolescent/M	Multiple cavitary lesions	Positive	Diagnostic	Steroids + rituximab	Improved
Monroe et al. [11]	2025	NR/M	GPA masquerading as tuberculosis	Positive	Diagnostic	Immunosuppression	Improved
Present case	2025	33/M	Hemoptysis, large cavitary lesion	C-ANCA+/PR3+	Nondiagnostic transbronchial biopsy	Steroids + rituximab	Improved

This case highlights an uncommon presentation of GPA in which isolated pulmonary disease masqueraded as an infectious cavitary process. Early recognition of vasculitis in the differential diagnosis of cavitary lung disease, coupled with timely initiation of immunosuppressive therapy, is essential to prevent disease progression and irreversible organ damage.

## Conclusions

Granulomatosis with polyangiitis should remain an important consideration in patients presenting with cavitary pulmonary lesions, particularly when infectious and malignant etiologies have been excluded. This case highlights an uncommon presentation of GPA characterized by isolated pulmonary involvement, hemoptysis, and a large cavitary lesion in the absence of classic upper airway or renal manifestations. The diagnosis was established through integration of clinical, radiographic, and serologic findings despite nondiagnostic transbronchial biopsy results. Review of previously reported cases demonstrates that cavitary pulmonary GPA frequently mimics more common infectious processes, contributing to diagnostic delays. Early recognition and prompt initiation of immunosuppressive therapy, including rituximab-based regimens, are critical to achieving remission, preventing irreversible organ damage, and improving long-term outcomes.
